# Novel application of live imaging to determine the functional cell biology of endothelial-to-mesenchymal transition (EndMT) within a liver-on-a-chip platform

**DOI:** 10.1007/s44164-022-00034-9

**Published:** 2022-09-20

**Authors:** James Whiteford, Samantha Arokiasamy, Clare L. Thompson, Neil P. Dufton

**Affiliations:** Barts and the London School of Medicine and Dentistry, William Harvey Research Institute, Queen Mary University of London, London, UK

**Keywords:** EndMT, Fibrosis, Organ-on-a-chip, Vascular biology

## Abstract

**Objective:**

Imaging endothelial cell behaviour under physiological conditions, particularly those associated with chronic fibrotic pathologies, is an incredibly challenging endeavour. While short-term assessments (hours) can be achieved with techniques such as intravital microscopy, vascular changes often occur over days and weeks which is unfeasible with current imaging techniques. These challenges are exemplified within the liver where liver sinusoidal endothelial cells (LSECs) are known to undergo dramatic changes termed endothelial-to-mesenchymal transition (EndMT) during fibrotic liver disease. Despite the established presence of EndMT in liver disease, the inaccessibility of viable liver tissue, and simplicity of 2D culture techniques has meant, the role of EndMT during disease progression remains largely undetermined. This study describes the development of novel fluorescent EndMT reporters to identify, track, and characterise the migratory behaviour of EndMT cells. We show that liver-on-a-chip (LOAC) platforms provide a flexible, optically accessible, and physiologically relevant microenvironment to study the vascular dynamics of EndMT during liver disease.

**Methods:**

Identification, creation, and application of an EndMT-specific fluorescent reporter construct (EndMT-Rep). Transduction of EC using lentiviral packaged CNN1-eGFP construct as an inducible EndMT-Rep (CNN1-Rep) to 2D, 3D, and 4D imaging techniques for fixed and live cell imaging. Combined application of live and fixed imaging technologies to measure EndMT using CNN1-Rep on LOAC platform under physiological conditions. Demonstration of the high-resolution single-cell EndMT tracking by live cell time-lapse microscopy and with post-acquisition processing to perform a comparative study of CNN1-Rep and healthy LSECs within a NASH-like LOAC microenvironment.

**Conclusions:**

LOAC enables prolonged, multi-platform imaging of endothelial cell sub-populations such as those undergoing EndMT in 2D and 3D cultures. Our study highlights the application of EndMT reporters, such as CNN1-Rep, to provide high-resolution imaging of EndMT behaviour for the first time under physiologically relevant liver microenvironment. Overall, these methods reveal the adaptability and impact of live-cell imaging on uncovering vascular behaviours, such as EndMT, that are unattainable in viable tissue or conventional 2D in vitro experiments.

**Supplementary Information:**

The online version contains supplementary material available at 10.1007/s44164-022-00034-9.

## Background

**Fig. 1:**
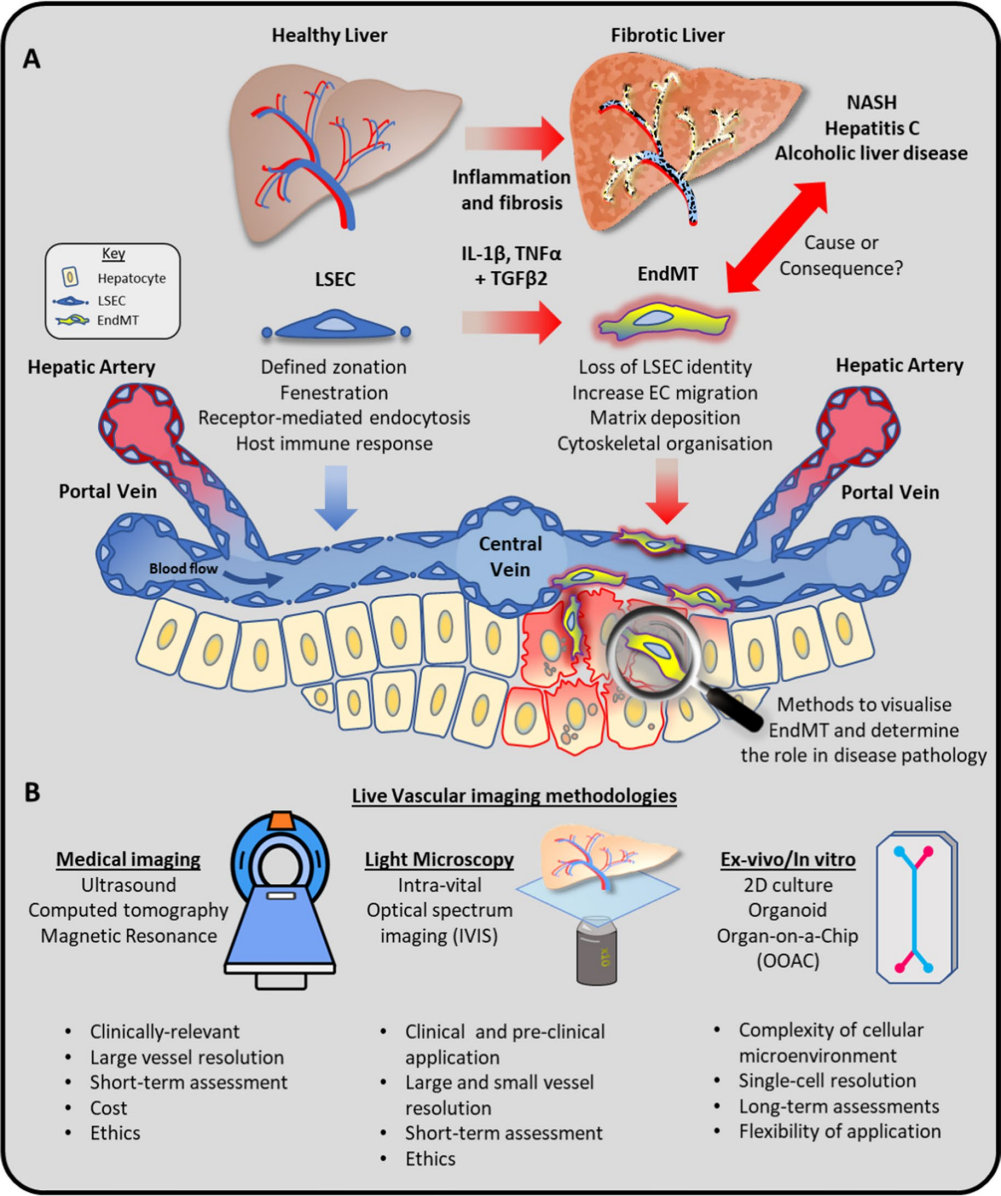
**A** Schematic of the of vascular changes that occur in response to inflammatory and profibrotic microenvironments leading to progressive liver diseases such as non-alcoholic steatohepatitis (NASH) and alcoholic liver disease and viral infections exemplified by hepatitis C. Elevated levels of the pro-fibrotic factor TGFβ2 drive a loss of liver sinusoidal endothelial cell (LSECs; blue) identity and acquisition of mesenchymal genes expression in a process termed endothelial-to-mesenchymal transition (EndMT; yellow) which is further synergised in the presence of inflammatory mediators (IL1-β and TNFα). While the LSECs are uniformly distributed throughout the liver forming a lattice between hepatocytes to facilitate efficient interactions between circulating blood and hepatocytes, EndMT is associated with disruption of hepatic architecture through increased matrix deposition and changes in vascular organisation. While EndMT is a well-established trait of fibrotic liver disease, their contribution to disease progression and potential for therapeutic intervention is a hotly debated topic. **B** There are a range of imaging technologies available to assess vascular integrity in liver tissue ranging from medical imaging applicable in the clinic to light microscopy and ex-vivo/in vitro techniques that are more available for basic research. We discuss some of the benefits and caveats to each approach and highlight the exciting new potential for multi-cellular, 3D liver-on-a-chip providing unrivalled access for imaging vascular changes such as EndMT that are not currently possible in patients or pre-clinical in vivo models

The adaptability and plasticity of blood vessels, and particularly the cells that line them, endothelial cells (EC), are fundamental for our body’s rapid response to tissue injury and infection. Every vascular bed of the body must develop and maintain unique adaptations to facilitate efficient gas and nutrient exchange in dramatically different tissue microenvironments. Specialised vessels such as arteries, veins, and capillaries are well-established examples of different vascular identities that seamlessly integrate to form our circulatory system. In recent years, single-cell RNA sequencing (scRNAseq) has provided incredible insight into whole organism and organotypic cellular heterogeneity, revealing that ECs are amongst the most diverse and plastic of all cell types in the body. The liver exemplifies the astonishing breadth of EC specialisation with approximately 7 distinct EC subpopulations in healthy tissue [1]. Notably, during the initiation and progression of liver disease, liver sinusoidal endothelial cells (LSECs) undergo dramatic changes to their transcriptomic profiles, which have been strongly associated with the pathophysiology of pre-clinical murine models of liver injury[2] and correlate with vascular changes in patients with alcoholic liver disease[3] and non-alcoholic steatohepatitis (NASH)[4, 5]. The mechanisms that regulate these phenotypic changes have come to the forefront of vascular research with the most well-established process termed endothelial-to-mesenchymal transition (EndMT). EndMT defines the loss of EC characteristics and acquisition of mesenchymal-like genes driven primarily by TGFβ canonical signalling and synergised by inflammatory cytokines including TNFα and IL-1β (Fig. 1A). The transcriptional features of EndMT are highly conserved between tissues and mammalian species making it an intriguing target for pre-clinical and translational studies of fibrosis.

One of the major challenges in determining the impact of these dramatic vascular changes is the ability to conduct detailed imaging studies within a viable liver tissue (Fig. 1B). Despite huge strides in the application of non-invasive, deep-tissue imaging technologies, such as ultrasound, computed tomography, and magnetic resonance, their use to observe vascular change in the liver is still in their infancy[6, 7]. While they provide great insight into the overall changes in hepatic architecture, they currently do not provide the microvascular resolution required to assess processes such as EndMT in animal models or patient tissue. Furthermore, they are constrained by relatively short-term application (a few hours) and incur prohibitive costs restricting their application to pre-clinical assessments. More invasive techniques such as IVIS[8] and intravital microscopy[9] can provide significantly enhanced microvascular resolution and even enable single-cell imaging. However, they do pose several practical challenges including being technically demanding and involve regulatory and ethical considerations required for in vivo experimentation. The most accessible approaches for single-cell assessment of endothelial behaviour are in vitro culturing techniques, although the practical benefits have been largely outweighed by their poor representation of physiological microenvironments. These stigmatisations have been challenged by the rapid development of three-dimensional, multicellular culture techniques exemplified by organoids and the application of more adaptable biomaterials and 3D printing technologies. Together, these advances have resulted in a huge increase in commercially available organ-on-a-chip (OOAC) platforms. Liver-on-a-chip (LOAC) technologies have been successfully employed to address the issue of hepatotoxicity, which is the most common cause of failure of phase 1 drug trials. These systems incorporate multiple species specific–cell types, vasculature, and immune components to generate models with greater predictive power which enable long-term pharmacokinetic studies[10, 11]. In this methodology study, we utilise ‘The Human Emulation System’ from Emulate Inc. to highlight how the accessibility and flexibility of LOAC platforms provides unrivalled opportunities to apply advanced molecular reporters to conduct high-resolution live imaging of healthy and transitioning EndMT cells for the first time.

## Validation and generation of EndMT-specific lentiviral reporters

**Fig. 2:**
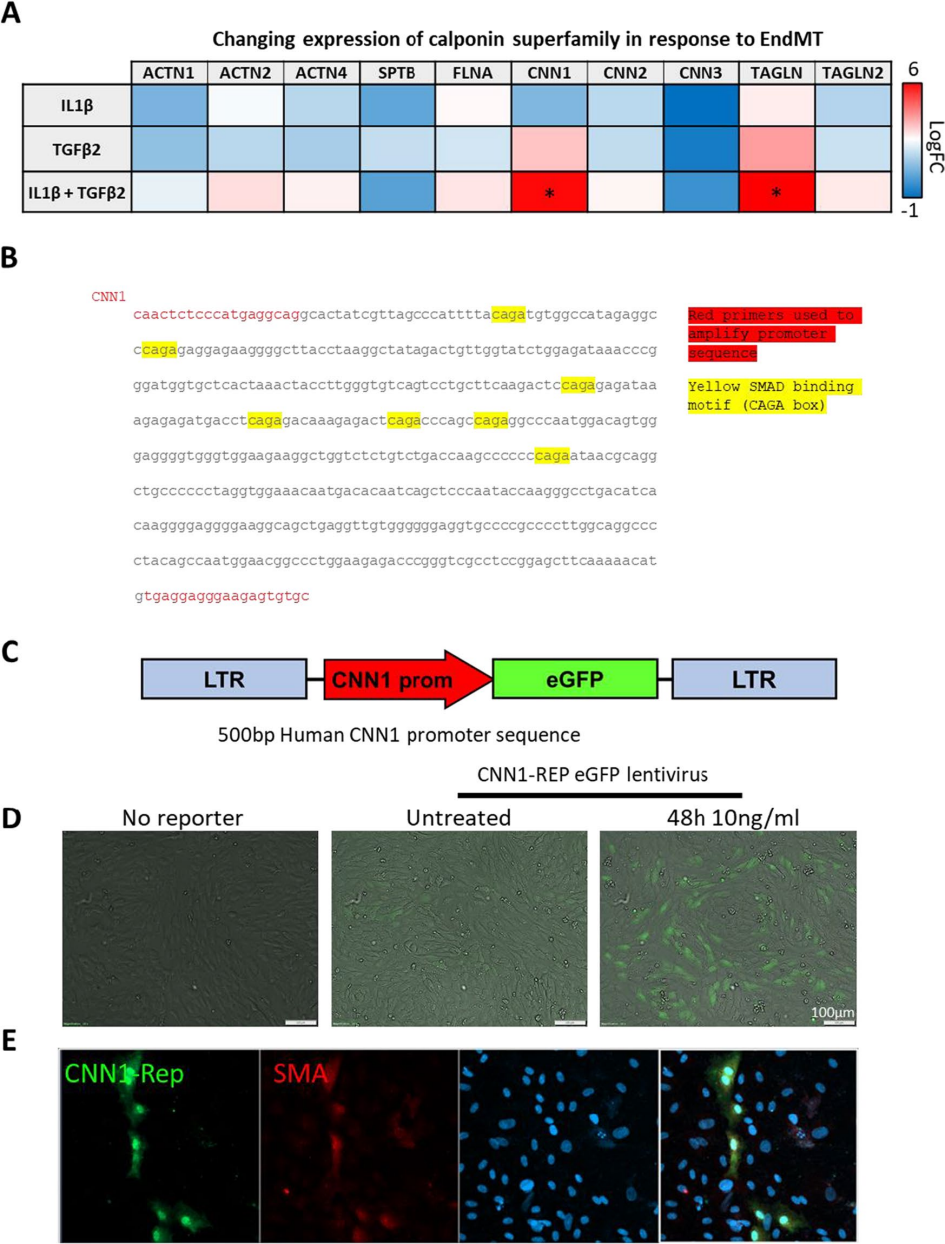
Meta-analysis of a published scRNAseq study. [12] revealed that a transcriptional distinct EndMT phenotype was induced in vitro by IL1β + TGFβ2, over 7 days. **A** Heatmap reveals transcriptional changes in members of the calponin superfamily of actin binding proteins with the most significant fold change increase compared to untreated controls or IL1β and TGFβ2 alone observed for calponin 1 (CNN1; 6.3-fold) and transgelin (TAGLN; 6.4-fold). **B** Promoter region (500 bp upstream of TSS) of human CNN1 annotated to show the primer sequences (red) and abundance of putative SMAD binding elements (CAGA boxes; yellow). **C** Schematic of eGFP CNN1-reporter construct designed by encoding CNN1 cDNA promoter sequence with eGFP and inserted into lentiviral vector plentiSFFV-MCS. **D** HUVEC cultured in a 24-well plate, transduced with viral preparations of either control or CNN1-Rep, were treated with 10 ng/ml TGFβ2 for 48 h and eGFP CNN1-Rep signal was imaged by live cell imaging Olympus iX82. E) CNN1-Rep transduced HUVEC treated with 10 ng/ml TGFβ2 for 48 h were co-stained with SMA (Red) and DAPI (Blue)

Several scRNAseq in human and rodents have revealed striking commonalities in the transcriptional programmes induced by EndMT. Gene ontology (GO) analysis of up-regulated genes have identified strong association of EndMT genes with immune response, cell migration, and cytoskeletal genes[12]. We chose to focus on members of the calponin superfamily of actin binding proteins that are amongst the most highly upregulated (Fig. 2A) and conserved in human and mouse (Fig. 2B). Calponin-1 (CNN1)[3, 12, 13] and transgelin (TAGLN or SM22-α)[12–14] are amongst the most widely cited EndMT genes with strong induction by TGFβ2 with the promoter regions containing a multitude of putative SMAD binding elements, known as CAGA boxes[15] (Fig. 2B). Due to these features, we chose the CNN1 promoter to develop an EndMT reporter construct. DNA (535 bp immediately preceding the ATG start codon) corresponding to the promoter sequence upstream of the human CNN1 gene was amplified by PCR from genomic DNA isolated from HEK 293 T cells and ligated into the *EcorI* and *BamHI* sites of the lentiviral backbone plasmid pLNTMCS-eGFP using standard procedures[16]. Lentiviruses were packaged in HEK 293 T cells (cultured in DMEM, 10% FBS and 1% penicillin) under the control of the GagPol promoter using the VSVG envelope protein. After 24-h cell growth, the medium was replaced. Supernatant containing viruses was collected 72-h post-transfection, loaded into a 20-ml syringe and filtered through a 0.45-µm filter. Pellets were resuspended in 1/10 of the initial supernatant volume in OptiMEM, and aliquots (100 µl) were stored at − 80 °C. Transduction of HUVECs was performed by the addition of viral preparations to cultured cells followed by incubation overnight. Transfection efficiency was > 90% measured by the induction of CNN1-Rep after 72 h treatment with TGFβ2 in HEK-293 cells (Sup Fig. 1). To validate whether this reporter was able to determine the induction of EndMT in live EC, HUVECs were transduced with CNN1-eGFP reporter (CNN1-Rep) for 24 h prior to treatment with media alone or 10 ng/ml TGFβ2 for 48 h. While we observed some basal activity of CNN1-Rep in untreated HUVEC, it was strongly upregulated by TGFβ2 by imaging either live cell (Olympus iX81 epifluorescent microscope; Fig. 2D) or post-fixation with 1% paraformaldehyde (PFA) for 15 min and co-staining with SMA as standard marker for EndMT (Zeiss LSM 800 confocal microscope; Fig. 2E). Together, these data show that CNN1-Rep is an EndMT-specific marker in live and fixed ECs and can be applied as a robust method to track transitioning EndMT cells in vitro.

## High-resolution imaging of EndMT using CNN1-Rep within liver-on-a-chip platform

**Fig. 3:**
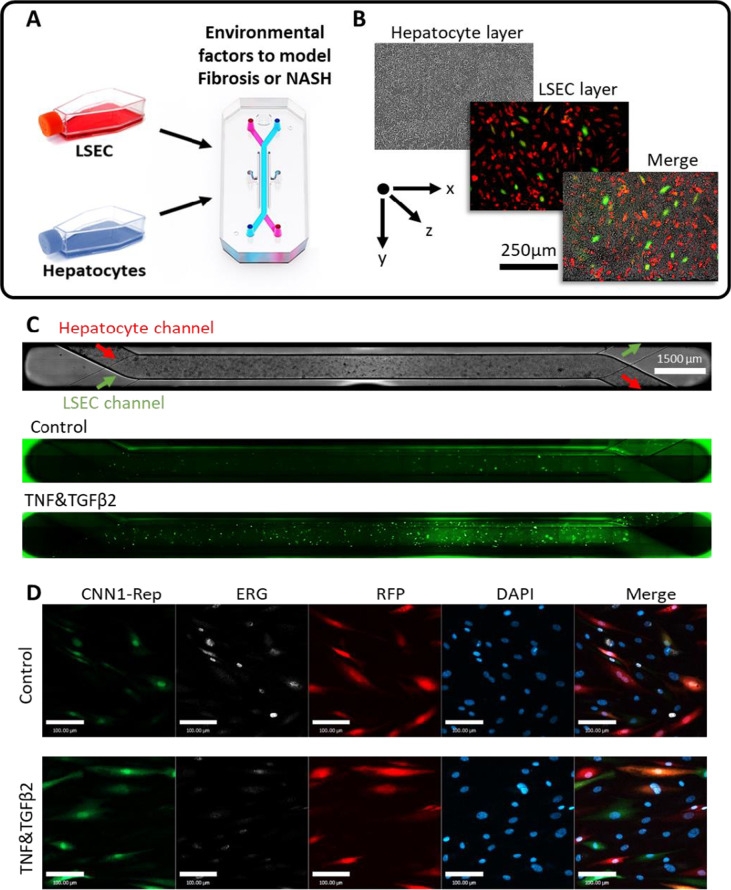
**A** Schematic of liver-on-a-chip (LOAC) co-culture using human LSECs and HepG2 human hepatocyte cell line. LSECs were co-transduced with CNN1-Rep and constitutive RFP reporter 24 h prior to seeding on LOAC. Cells were allowed to acclimatise under flow for 24 h prior to treatments being added to media reservoirs for 24–72 h. **B** Treated LOAC were transferred to MuviCyte (Perkin Elmer) in incubator fluorescent microscope to obtain brightfield and multichannel z-stack images of LSECs and hepatocyte layers. **C** MuviCyte software enables programmable single-field imaging and stitched whole LOAC imaging. CNN1-Rep reporter allows live imaging and quantification of whole LOAC induction of EndMT following 48 h of 10 ng/ml TGFβ2 and 1 ng/ml TNFα. **D** Whole mount imaging captured by confocal microscopy (Zeiss LMS 800) of fixed cells allows high-resolution comparison of CNN1-Rep^+^ LSECs with healthy RFP^+^ LSECs while counterstaining with ERG (white) and DAPI (blue) confirm TGFβ2 + TNFα induction of CNN1-Rep.^+^ EndMT (scale 100 µm)

LOAC were prepared using the Chip S1® (Emulate Inc.) in line with manufacturer’s protocols[10, 17]. Manufactured from poly-dimethylsiloxane (PDMS), this microfluidic chip comprises two overlapping channels separated by a porous membrane (7 µm diameter). Briefly, the PDMS surfaces of the apical and basal channels were first activated with ER-1 solution (Emulate Inc) and then exposed to UV irradiation for 10 min. This process was repeated, and then channels are washed sequentially with ER-2 (Emulate Inc) then PBS (Sigma Aldrich). Chips were then coated with collagen type I (100 µg/ml) and fibronectin (25 µg/ml) at 4 °C for 24 h prior to seeding liver sinusoidal ECs (LSECs; ScienCell) and hepatocyte cell line (HepG2), which were chosen as an accessible cell line for our proof-of-concept LOAC co-culture platform. HepG2 cells were first seeded in the apical channel (day 0) and allowed to adhere for 3 h prior to washing and overnight culture. LSECs were seeded in the basal channel on day 1 and the chips immediately inverted for 2 h to encourage attachment to the underside of the membrane. Chips were then returned to an upright position and cultured for a further 2 h prior to washing and overnight culture. After 24-h pre-culture, the LOAC were inserted into the Pod®, a portable media reservoir, then connected to the automated cell culture module, ZÖE (Emulate Inc.), and cultured under flow (30 µL/hour) for a further 24 h prior to the start of the experiment (schematic Fig. 3A). Prior to seeding, LSECs were transduced for 24 h with a combination of constitutive RFP reporter and EndMT reporter CNN1-Rep. Images of each cellular layer and reporter were then acquired at baseline (days 2/3 post-seeding) and 48 h post-incubation with TGFβ2 and TNFα (10 ng/ml and 1 ng/ml respectively; days 4/5 post-seeding) to induce EndMT using the MuviCyte Live-Cell Imaging System (PerkinElmer, Waltham, MA, USA). The MuviCyte allowed us to capture both high-resolution single frame (Fig. 3B) and tiled (Fig. 3C) images detecting brightfield and fluorescent channels to distinguish between hepatocytes, healthy LSECs (red), and EndMT LSECs (green; Fig. 3B). The incorporation of the Pod imaging adapter (Emulate Inc[18]) with the automated stage of the MuviCyte allowed widefield tiled scan images of the whole LOAC to be obtained in brightfield and fluorescence for CNN1-Rep at baseline and 48 h post-treatments (Fig. 3C). On day 7, LOAC was perfused with 1% PFA for 30 min and stained as a whole mount preparation to image the transduced EC reporters in combination with endothelial transcription factor ERG (Abcam, ab92513, 1:100 dilution overnight) and donkey-anti-rabbit 647. In line with our previous research, we showed that the induction of EndMT, seen by increased expression of CNN1-Rep, was stable post-fixation and mirrored by the reduction of the homeostatic EC-specific transcription factor ERG[3] using Zeiss LSM 800 confocal microscope.

## Quantification of dynamic EndMT behaviour in healthy and fibrotic LOAC microenvironments

Single cell analysis of EndMT in vitro, in vivo, and our current studies in LOAC all observe morphological changes (Fig. 3D) in response to EndMT. We hypothesised that the induction of cytoskeletal genes of the calponin superfamily and their morphological changes indicated that EndMT are more migratory than healthy LSECs. To test this hypothesis, we developed a live imaging time-lapse methodology to track EndMT and compare their migratory behaviour with healthy LSECs while modelling a disease microenvironment.

**Fig. 4:**
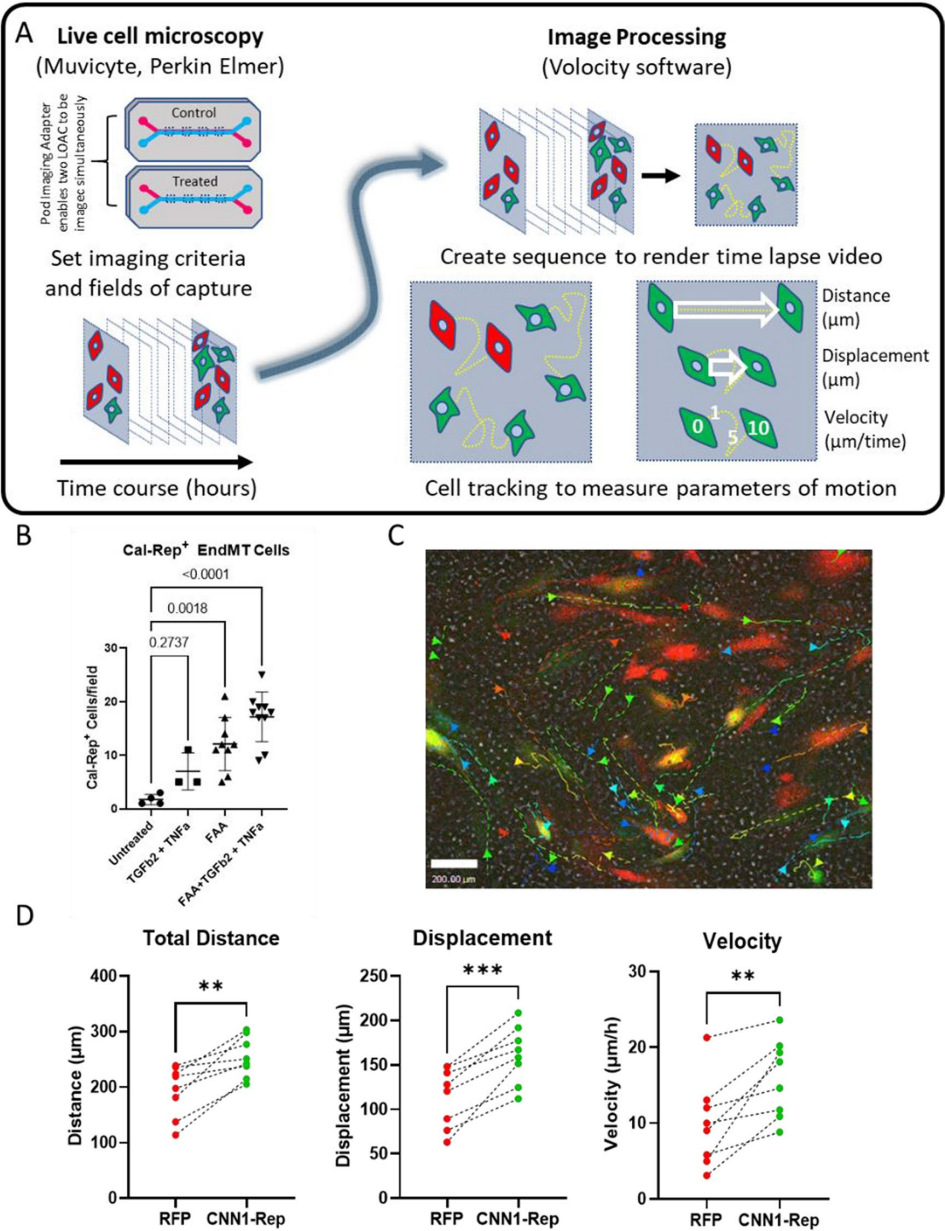
**A** Schematic of time-lapse live cell imaging of LOAC. The *‘*Pod imaging adapter’ for the automatic MuviCyte stage enables accurate field acquisition for 2 LOACs at the same time, of multiple regions of interest either as individual areas or stitched images in single depth or using Z-stack (recommended) in Tiff file format. MuviCyte image tools (PerkinElmer) enables repetitive imaging of each field at high frequency (Z-stack of 10 images with 3 channels ~ 5 min; therefore, one image cycle 4 fields for 2 LOACs requires ~ 1 h between each allotted time point). Once all fields and time points are captured, image files were transferred to Volocity software to create a first a multichannel Z-stack and then a time-course sequence to generate time-lapse videos. Volocity software provides modular plugins to identify objects, filter by size, and track by frame providing extensive single-cell tracking data. For migration studies, we have focused on the parameters of total distance travelled (μm), displacement (μm), and velocity (μm/time) for either individual or meaned datasets per field. **B** Static quantitation of CNN1-Rep^+^ EndMT induction between treatments. **C** Still from 24-h time-lapse imaging showing of individual cell tracking data for CNN1-Rep^+^ EndMT cells following 48-h treatment with a combination of FAA and TGFβ2 + TNFα to model NASH-like microenvironment. **D** By performing quantitative cell tracking in both ‘healthy’ constitutive RFP^+^ LSECs and eGFP CNN1-Rep^+^ EndMT LSECs with, we evaluated the migratory behaviour of these different cell populations under the same environmental conditions per field (*n* = 8 constituting 4 fields on 2 separate LOAC). **P < 0.01 or ***P < 0.001 by unpaired *T*-test between groups

The pod imaging adaptor enabled us to image two LOAC in parallel on the MuviCyte while the MuviCyte Image Tools software allowed us to perform Z-stack at multiple fields over 24 h. Imaging a 10 frame Z-stack in 3 channels (Brightfield, 488 and 555) took approximately 5 min per field; 4 fields per LOAC took 45–50 min enabling us to undertake imaging of each field every hour generating > 100 GB of imaging data when captured using TIFF format. To render videos from these individual time points, we exported each z-stack to Volocity imaging software (PerkinElmer) to produce a project of each time point. Each projection was then collated into a second image sequence, this time organised by time to generate a time-lapse video of cellular migration over time. Finally, built in Volocity algorithms were applied to ‘Identify Objects’ and ‘Track’ with adjustable setting enhancing contrast and differentiate between cells. We applied these parameters to EndMT CNN1-Rep^+^ cells and healthy RFP^+^LSECs to track the distance, displacement, and velocity (summarized in Fig. 4A). Together, our image processing enabled us to measure the induction and migration of LSECs under different physiological conditions revealing that LSECs within a NASH-like microenvironment of circulating free fatty acid and pro-fibrotic environment[19] modelled in our experiments by TGFβ2 and TNFα were most prone to undergoing EndMT (Fig. 4B). We therefore used these conditions as proof-of-concept to conduct in-depth cell tracking analysis of CNN1-Rep and RFP-LSECs demonstrating our strategy of cell tracking (Fig. 4C and supplementary videos) and reveal that CNN1-Rep^+^ EndMT have an enhanced migratory phenotype compared to healthy LSECs (Fig. 4D). All data generated or analysed during this study are included in this published article.

## Conclusions

The validation of an EndMT-Rep tool as a measure of tissue fibrosis in OOAC platforms will have a significant impact on our understanding of how vascular plasticity and dysfunction effect local tissue environments such as within the liver. Non-invasive assessment of vascular dysfunction, such as EndMT, has considerable challenges in animals and humans due to the depth, motion, and lack of resolution. This methodology overcomes these significant challenges and demonstrates the first study to perform high-resolution imaging and quantification of the single cell dynamics of EndMT in real-time. We believe these approaches highlight the untapped potential of OOAC platforms in bridging our understanding of vascular dysfunction observed in pre-clinical animal models and patients in addition to providing an unrivalled window to determine the functional impact of these changes.

Beyond the liver-specific application detailed in this study, EndMT has been established to occur in numerous chronic pathologies including pulmonary hypertension, renal, and cardiac fibrosis. The continued development and application of cell reporters to OOAC platforms could have wide-ranging implications from replacing invasive imaging in animal models to advancing personalised medicine by providing physiological environments to assess the function of patients’ cells as diagnostic, prognostic, or drug-screening assays to combat chronic fibrosis disease.

## Supplementary Information

Below is the link to the electronic supplementary material.

Supplementary file1 (DOCX 1378 KB)

Supplementary file2 (AVI 9003 KB)

Supplementary file3 (AVI 9003 KB)

Supplementary file4 (MP4 11991 KB)

## Data Availability

The datasets generated during the current study are available from the corresponding author on reasonable request.
