# Supplementary material for: Novel application of live imaging to determine the functional cell biology of endothelial-to-mesenchymal transition (EndMT) within a liver-on-a-chip platform
Source: In Vitro Model. 2022 Sep 20;1(6):413–21. doi: 10.1007/s44164-022-00034-9 (PMC9767233; doi:10.1007/s44164-022-00034-9)
Supplement: Supplementary file 1 — Supplementary file1 (DOCX 1378 KB) [file 44164_2022_34_MOESM1_ESM.docx]

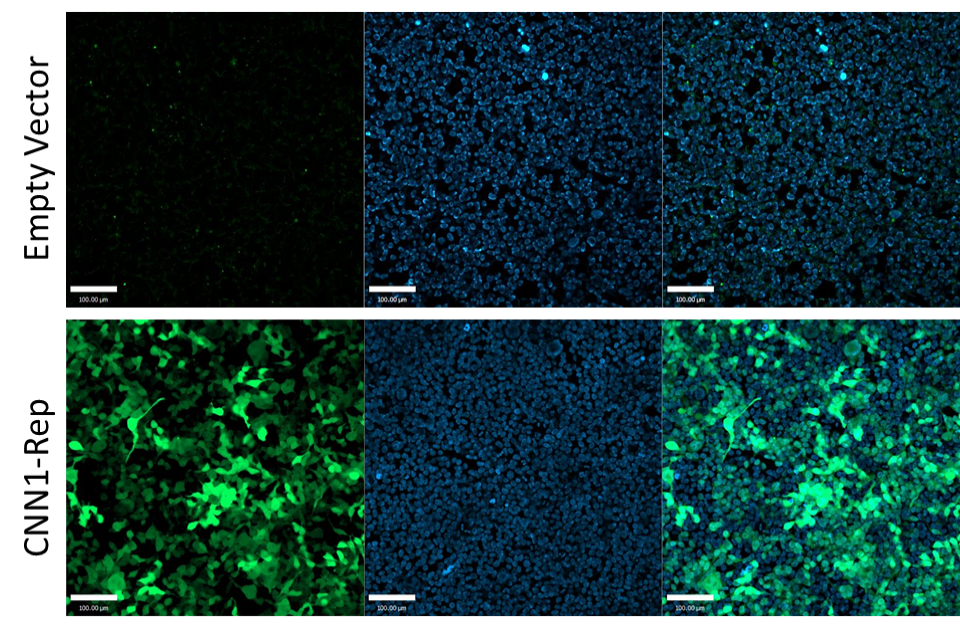
**Supplementary Figure 1.**

***Supplementary Figure 1.*** *Representative image of HEK-293T Cells transduced for 24 hours with empty vector or CNN1-Rep. Cells were treated with 10 ng/ml TGFβ2 for 72 hours.*

**Supplementary Videos 1.** Representative video of control untreated LSECs *transduced* with CNN1-Rep (green) 24 hours prior to seeding on LOAC. Hepatocyte cell layer are false coloured red for contrast.

**Supplementary Videos 2.** Representative video of LOAC perfused with TGFβ2 (10 ng/ml) and TNFα (1 ng/ml) for 48 hours prior to imaging. LSECs were *transduced* with CNN1-Rep (green) 24 hours prior to seeding on LOAC. Hepatocyte cell layer are false coloured red for contrast.

**Supplementary Videos 2.** Video for still image Fig. 4C showing tracking of CNN1-Rep^+^ EndMT cells and RFP LSECs in the same field. LOAC was perfused with Free Fatty Acids (Palmitic and Oleic acid; 0.3 and 0.6mM respectively) in combination with TGFβ2 and TNFα for 48 hours prior to imaging to model NASH-like environment. LSECs *transduced* with CNN1-Rep (green) and constitutive RFP (red) in combination 24 hours prior to seeding on LOAC.
